# A Sweetpotato Geranylgeranyl Pyrophosphate Synthase Gene, *IbGGPS*, Increases Carotenoid Content and Enhances Osmotic Stress Tolerance in *Arabidopsis thaliana*

**DOI:** 10.1371/journal.pone.0137623

**Published:** 2015-09-16

**Authors:** Wei Chen, Shaozhen He, Degao Liu, Gunvant B. Patil, Hong Zhai, Feibing Wang, Troy J. Stephenson, Yannan Wang, Bing Wang, Babu Valliyodan, Henry T. Nguyen, Qingchang Liu

**Affiliations:** 1 Beijing Key Laboratory of Crop Genetic Improvement/Laboratory of Crop Heterosis and Utilization, Ministry of Education, China Agricultural University, Beijing, China; 2 Division of Plant Sciences and National Center for Soybean Biotechnology, University of Missouri, Columbia, Missouri, United States of America; Nanjing Agricultural University, CHINA

## Abstract

Sweetpotato highly produces carotenoids in storage roots. In this study, a cDNA encoding geranylgeranyl phyrophosphate synthase (GGPS), named *IbGGPS*, was isolated from sweetpotato storage roots. Green fluorescent protein (GFP) was fused to the C-terminus of IbGGPS to obtain an IbGGPS-GFP fusion protein that was transiently expressed in both epidermal cells of onion and leaves of tobacco. Confocal microscopic analysis determined that the IbGGPS-GFP protein was localized to specific areas of the plasma membrane of onion and chloroplasts in tobacco leaves. The coding region of *IbGGPS* was cloned into a binary vector under the control of 35S promoter and then transformed into *Arabidopsis thaliana* to obtain transgenic plants. High performance liquid chromatography (HPLC) analysis showed a significant increase of total carotenoids in transgenic plants. The seeds of transgenic and wild-type plants were germinated on an agar medium supplemented with polyethylene glycol (PEG). Transgenic seedlings grew significantly longer roots than wild-type ones did. Further enzymatic analysis showed an increased activity of superoxide dismutase (SOD) in transgenic seedlings. In addition, the level of malondialdehyde (MDA) was reduced in transgenics. qRT-PCR analysis showed altered expressions of several genes involved in the carotenoid biosynthesis in transgenic plants. These data results indicate that *IbGGPS* is involved in the biosynthesis of carotenoids in sweetpotato storage roots and likely associated with tolerance to osmotic stress.

## Introduction

Carotenoids are widely produced in many plants and provide potent nutritional benefits to human and animal health. The biosynthetic pathway of carotenoids has gained relatively intensive investigations in different plants [1, 2]. Carotenoids are mainly biosynthesized from the MEP pathway through isopentenyl diphosphate (IPP), dimethylallyl diphosphate (DMAPP), geranyl pyrophosphate and geranylgeranyl pyrophosphate (GGPP) in plastids [1, 3]. Two GGPP molecules are condensed to a linear C40 backbone towards different structures of carotenoids, such as beta-carotene and zeaxanthin.

The formation of GGPP is a key step in biosynthetic pathway of carotenoids and many other terpenes. This step is catalyzed by geranylgeranyl pyrophosphate synthase (GGPS) [3, 4]. *GGPS* cDNAs have been cloned from multiple plant species. A gene expression study using sunflower seedlings showed that the *HaGGPS* was expressed after 2 days of seed imbibition [5]. The expression of *GGPS* cloned from *Ipomoea sp*. was found to change dramatically during flower development [6]. Recently, the expression of *GGPS* in sweet orange was characterized to be regulated by a miRNA [7]. In addition, the expression of *GPPS* has been found to associate with resistance of plants to different stresses. The silencing of *NaGGPS* in *Nicotiana attenuate* was found to decrease resistance to hornworm [8]. The expression of GGPS in *Arabidopsis thaliana* has been found to associate with protection against photo-oxidative stress [9]. In addition, molecular and function characterization of GGPS has been reported in multiple plant species, such as *Taxus canadensis* [10], *A*. *thaliana* [11, 12], *Scoparia dulcis* and *Croton sublyratus* [13], *Helianthus annuus* [5], *Gentiana lutea* [14], *Hevea brasiliensis* [15] and *Solanum lycopersicon* [16]. Furthermore, A *GlGGPS* cDNA from *G*. *lutea* was used to rescue a *ggps E*.*coli* mutant [14], indicating a functional conservation.

Sweetpotato (*Ipomoea batatas* (L.) Lam.) is an important crop not only for food products but also for numerous other by-products [17]. Sweetpotato storage roots provide multiple benefits to human health, e.g. antioxidative activity resulting from a high production of carotenoids [18, 19]. To date, carotenoids become one of important agronomic traits to breed new varieties. To increase production, a few of genes have been cloned from sweetpotato, such as β-carotene hydroxylase (*CHY-β*) and lycopene ϵ-cyclase (*LCY*-ϵ) genes [20, 21]. In addition to nutritional benefits, carotenoids were recently found to increase sweetpotato resistance to stress conditions [20]. A down-regulation of *CHY-β* gene was observed to increase the contents of β-carotene and total carotenoids in cultured transgenic sweetpotato cells. It was interesting that those transgenic cells showed higher salt stress tolerance than wild-type control cells [20]. Besides, Down-regulation of *LCY*-ϵ gene increased carotenoid synthesis and enhanced salt-stress tolerance in transgenic calli of sweetpotato [21].

To understand carotenoid biosynthesis in sweetpotato, we report the cloning and transgenic analysis of sweetpotato *GGPS*. A cDNA namely *IbGGPS* was cloned from the storage roots of sweetpotato. Its full length of cDNA was fused GFP to localize protein in cells. Its overexpression in *A*. *thaliana* increased the contents of total carotenoids and tolerance of transgenic plants to osmotic stresses.

## Materials and Methods

### Plant materials

Sweetpotato (*Ipomoea batatas* (L.) cv. Nongdafu 14 containing high concentrations of carotenoids [22] was employed for gene cloning in this study. Plants were grown in a field at Langfang Experimental Station, Hebei, China. Storage roots were collected when the diameter was 4–5 cm, frozen immediately in liquid nitrogen and then stored at -80°C prior to use *A*. *thaliana* (ecotype Columbia-0, Col-0) was grown following the standard growth protocol (published on tair).

### Cloning of *IbGGPS* gene

Total RNA was extracted from storage roots using the Trizol reagent (Invitrogen, Carlsbad, CA, USA) and then subjected to a DNase treatment. The quality and concentration of the extracted RNA were examined by agarose gel electrophoresis and by spectrophotometer (DU-640, Beckman, Brea, CA, USA) analysis. The RNA samples were stored at -80°C prior to rapid amplification of cDNA ends (RACE) and reverse transcription polymerase chain reaction (RT-PCR) analysis. The first strand cDNA was synthesized from 1 μg of RNA using Superscript II Reverse Transcriptase Kit (Invitrogen) and oligo (dT) primer according to manufacturer’s instructions. RT-PCR was performed using degenerated primers (DE-P-1: 5’-GARATGATHCAYACYATGTC-3’ and DE-P-2: 5’-TCYTTYCCDGCVGTYTTYCC-3’) that were designed based on the conserved sequence of other plants’ *GGPS* genes. Amplified product was cloned into a TA cloning vector and sequenced from both ends. The resulting sequence was used as a template to design primers to clone a full length *IbGGPS* gene by RACE-PCR. The 3’ and 5’ RACE Kit (Invitrogen) were used to amplify the 3’ and 5’ end of the *IbGGPS* gene. The primers used for 3’ RACE were: 3A (5’-GCGACGCTCTCCTCTCCTTC-3’), 3B (5’-GCCGGGCAAGTCGTCGATA-3’) and UPM (provided with the kit). The primers used for 5’ RACE were: 5A (5’-GTTTTTCCGAGCTCTTCAGACGATT-3’), 5B (5’-CGAATTTCCTCAGTTTCTCCACCTC-3’), 5C (5’-GATAAGAACTCGAAGGCGAAGGAGA-3’) and AUAP (provided with the kit) for the first round of PCR. The second round of PCR was performed usingIIR-PCR-1 (5’-CATATACGCCGTGAAATCAAAGCTC-3’), IIR-PCR-2 (5’-CACTAACCCTCACACCCTCTTCCTG-3’), IIR-PCR-3 (5’-GAACCGCCGAGACAGCAAAAC-3’) and AUAP primers. The PCR thermal cycle was composed of: 94°C for 3 min 180 s; 35 cycles of 94°C 30 s, 56°C 30 s, 72°C 120 s; followed by 72°C for 10 min and 4°C. The PCR products in gel were purified by following gel purification protocol. The resulting cDNA was cloned into pGEM-T Easy vector (Promega Co., Madison, WI, USA). The recombinant plasmid was transformed to *Escherichia coli* strain DH5α, which was subsequently plated onto Luria-Bertani (LB) plates supplemented with 50 μg ml^-1^ ampicillin, 50 mM isopropyl β-D-1-thiogalacto pyranoside (IPTG) and 244.72 mM X-gal. After 16 h of incubation at 37°C, white colonies growing on the plates were selected for screening by PCR using Primer SP6 (5’-ATTTAGGTGACACTATAG-3’) and Primer T7 (5’-TAATACGACTCACT ATAGGG-3’). The PCR thermal cycle was composed of 94°C for 5 min, followed by 35 cycles consisting of 94°C for 30 s, 60°C 30 s, 72°C 3 min and a final extension at 72°C for 10 min and 4°C. PCR products were examined by electrophoresis on a 2% (w/v) agarose gel. Positive colonies were identified for sequencing (Invitrogen, Beijing, China).

### Sequence analysis of *IbGGPS* gene

The cDNA sequence fragments were assembled and analyzed to obtain a full length of nucleotides using Seq-Man software. The assembled full length sequence was deduced to obtain amino acid sequences. The amino acid sequences of *A*. *thaliana*, *Medicago sativa*, *S*. *lycopersicum* and *Salvia miltiorrhiza* homologs were retrieved from NCBI GenBank database. Five sequences were aligned to compare sequence similarity using CLUSTAL-W.

### Subcellular localization of IbGGPS

A plasmid, namely pMDC83-*IbGGPS*-GFP, was constructed to analyze subcellular localization of IbGGPS. In brief, the coding sequence of *IbGGPS* was amplified through PCR using primers LOC1-F (5’-AAACTAGTATGAGGTCGATGAATCTTGT-3’, including a *Spe*I site underlined) and LOC1-R (5’-AAGGCGCGCCATTCTGTCTATAAGCTATGT-3’, including a *Asc*I site underlined). The pMDC83-GFP construct was used for IbGGPS and green fluorescent protein (GFP) fusion according to the method of Jiang et al. [23]. The amplified product was fused to the N-terminal of GFP under the control of cauliflower mosaic virus (CaMV) 35S to obtain a cassette consisting of 35S-IbGGPS-GFP. The resulting plasmid was named pMDC83-*IbGGPS*-GFP for bombarded transformation. The pMDC83-GFP construct was used as control. Two types of plasmids were bombarded into onion (*Allium cepa*) epidermal cells via microprojectile bombardment (PDS-1000/He, Bio-Rad, Hercules, CA, USA) with gold particles (1.0 μm) and a helium pressure of 1100 psi as described by Jiang et al. [23]. Bombarded onion epidermal cells were then incubated for 24 h at 25°C in the dark. The fluorescence of GFP was examined under a confocal fluorescence microscope.

In addition, pMDC83-*IbGGPS*-GFP and pMDC83-GFP plasmids were introduced to *Agrobacterium tumefaciens* EHA105 bacterial strain, respectively. The resulting positive colonies were used to infect leaf discs of *N*. *benthamiana* according to the method of Fu et al. [24]. Infected discs were incubated for 48 h at 25°C in the dark. Discs were washed well with autoclaved water to remove all *Agrobacterium* on the surface and then examined under confocal fluorescence microscope.

### Transformation of *Arabidopsis* with *IbGGPS* gene

A pMDC32-35S-*IbGGPS* binary vector was constructed for genetic transformation of *A*. *thaliana*. The coding sequence of *IbGGPS* was amplified using the GGPS-F (5'-CACCATGAGGTCGATGAATCTTGT-3') and GGPS-R (5'-TTCGTTAATTCTGTCTATAAGCTA-3') primers and cloned into the pENTR vector. The thermal cycle was composed of 94°C for 3 min; 35 cycles of 94°C 30 s, 60°C 30 s, 72°C 3 min; followed by 72°C for 10 min and 4°C. The resulting cDNA was cloned to the pMDC32 vector (Invitrogen) by LR recombination reaction following the manufacturer’s instructions. The recombinant vector (namely pMDC32-35S-*IbGGPS*) was electroporated into *Agrobacterium* strain GV3101 based on a reported protocol [23]. A positive pMDC32-35S-*IbGGPS*/GV3101 colony was identified and then used for agro-infiltration of *A*. *thaliana* inflorescences by following a protocol reported previously [25]. Putative transformed seeds were germinated on agar-solidified MS [26] medium containing 25 mg l^-1^ hygromycin (Hyg). Leaves of 3-week-old Hyg-resistant seedlings were harvested to isolate total RNA for PCR analysis. The first strand cDNA was synthesized as reported previously [27]. Primers including IbGGPS-TF (5'-ATGAGGTCGATGAATCTTGT-3') and IbGGPS-TR (5'-TTCGTTAATTCTGTCTATAAGCTA-3') were designed for PCR. The PCR product was checked by electrophoresis on a 1.5% (w/v) agarose gel. PCR-positive transgenic seedlings were grown in pots to select T_2_ and T_3_ seeds.

### Expression analysis for sweetpotato and transgenic *Arabidopsis*

Total RNA samples were isolated from storage roots, leaves and stems of sweetpotato using Trizol reagent. Meanwhile, total RNA samples were isolated from T_3_ transgenic vs wild-type (WT) *Arabidopsis* plants using the same method. The first strand cDNA was synthesized as described above. The *IbGGPS* expression in tissues of sweetpotato was analyzed using Real-time quantitative PCR (qRT-PCR). qRT-PCR was performed by ABI PRISM 7500 (Software for 7500 and 7500 Fast Real-Time PCR Systems, V2.0.1, USA) using SYBR qPCR Mix (Bio-Rad) reagents. Primer sets of 0.4 mM final concentration for each primer were used in a final volume of 20 μl. The primer sequences for qRT-PCR are listed in Table 1. Thermal profile of the qRT-PCR was at 95°C for 60 s, followed by 40 cycles of 95°C for 15 s and 60°C for 60 s. Dissociation curves were obtained using a thermal melting profile performed after the last PCR cycle: 95°C for 15 s followed by a constant increase in the temperature between 60°Cand 95°C. A 169 bp fragment of sweetpotato β-actin gene (Genbank AY905538), used as an internal control, was amplified by the specific primers (Table 1).

**Table 1:** Primers used in qRT-PCR and sqRT-PCR analyses.

Primer	Sequence (5’ to 3’)
**AtTUB4F**	CCGAAGGTGCTGAGTTGATT
**AtTUB4R**	TCCTCCCAATGAATGACACA
**AtZEPF**	CGGAGCTTTCTTCTTGATGG
**AtZEPR**	TCGATTTCGGAGTTTTCCTG
**AtPSYF**	GAAGACATATTCGCCGGAAA
**AtPSYR**	AGCAATGAAGCCCATACAGG
**AtPDSF**	CCAAACTGTGAACCATGTCG
**AtPDSR**	TTGCCTCCGACAACTTTCTT
**AtZDSF**	TAGCGATGCAGGTCACTGAG
**AtZDSR**	CTGATCGGGTCTGAATGGAT
**AtBCHF**	CAAGAGAAGGACCGTTCGAG
**AtBCHR**	GGAACGAGACCTTTGTGGAA
**IbGGPSRTF**	GGAAGAGGGTGTGAGGGTTAG
**IbGGPSRTR**	GCGGCGATACATAGCATGGG

The expression of *IbGGPS* in transgenic *Arabidopsis* was analyzed by semi-quantitative RT-PCR (sqRT-PCR). *Tubulin* gene was used as an internal control. PCR amplifications were performed as described above, and the PCR products were separated by electrophoresis on a 1% (w/v) agarose gel. The primer sequences are listed in Table 1.

Five carotenoid pathway genes of *Arabidopsis*, namely carotene hydroxylase (*AtBCH1*, NM_118702), phytoene synthase (*AtPSY1*, NM_121729), phytoene desaturase (*AtPDS*, NM_117498), carotene desaturase (*AtZDS*, NM_111359) and zeaxanthin epoxidase (*AtZEP*, NM_126103) genes, were analyzed in two transgenic vs. WT plants using qRT-PCR as described above.

### Seedling growth on PEG-infused medium

Polyethylene glycol (PEG)-infused (-0.7 MPa) plates were prepared as described by Verslues et al. [28]. Briefly, plates were made by dissolving solid PEG-8000 into a solution of basal MS medium and adjusting the pH to 5.7. This PEG solution was then added on the surface of an agar-solidified basal medium (3:2, v/v) in plates. All plates were placed on the bench at room temperature for 12 h, during which the PEG solution was immersed into the agar. The left excessive PEG solution on the top of agar was removed. The sterilized seeds of *Arabidopsis* WT and transgenic T_3_ plants were inoculated on the top of PEG-fused MS medium. All materials were also be cultured on MS medium without PEG as the control. Plates were placed vertically on a shelf for at 22°C under 16 h photoperiod supplied with cool-white fluorescent light at 50 μM m^-2^ s^-1^. After 10 days of germination of seeds, the root length of seedlings was measured.

### Extraction and quantification of carotenoids in transgenic *Arabidopsis*

Extraction and quantification of carotenoids were performed as described by Chen et al. [29]. Five hundred mg of fresh tissue was harvested into 6 ml of ethanol (96%, v/v) and shaken for 30 s, followed by heating for 5 min at 85°C. After heating, 500 μl of 80% KOH was added, and then the samples were vortexed for 30 s and heated for another 5 min at 85°C. Tubes were kept on ice and 3 ml of water and 3 ml of hexane were added. After 5 min of incubation, the samples were centrifuged for 1 min at 2,700 g and the supernatant was collected. The pellet was extracted twice with 3 ml of hexane and the supernatant was collected, pooled, and 3 ml of water was added. Tubes were shaken and centrifuged for 1 min at 2,700 g, and the organic phase was collected in a 20 ml glass tube. This step was repeated twice. The organic phase of each extraction was, pooled and dried under a stream of nitrogen gas. The residue was re-suspended in 500 μl of acetonitrile/methanol/ methylene chloride (40:25:35, v/v) and filtered through a 0.2 μm pore size nylon syringe filter. Twenty μl was used for HPLC analysis on LC2010HT (Shimadzu, Japan) equipped with a photodiode array detector. The column used was a 4.6 μm × 250 mm reverse-phase C30-YMC-Carotenoids Column (Waters Ltd, Missis-sauga, ON, Canada) and a placed in a 35°C chamber. The mobile phase used was composed of acetonitrile/methanol/methylene chloride (40:25:35, v/v) to form an isocratic elution system. Elutes were detected at 450 nm. Authentic standards (from Sigma-Aldarich, St. Louis, MO, US) were used to identify and quantify carotenoids.

### SOD and MDA measurements

Superoxide dismutase (SOD) activity was measured as described by He et al. [17]. First, 500 mg of fresh tissue was homogenized in 4 ml 50 mM cooled phosphate buffer (pH 7.8) containing 0.1mM EDTA. The homogenate was centrifuged for 15 min at 10,000 g and 4°C, and the supernatant was transferred to a new tube for enzyme assay as reported previously [30]. The malondialdehyde (MDA) measurement was performed according to Gao et al. [30]. Fresh tissue (500 mg) was ground with 10 ml 10% (w/v) trichloroacetic acid (TCA) on ice. The homogenate was centrifuged at 4,000 g for 10 min at 4°C, and the supernatant was transferred to a new tube. Two ml of 0.6% thiobarbituric acid (TBA) were added to 2 ml of the supernatant in a 10 ml tube. The tube containing the mixture was kept in boiling water for 15 min and placed on ice to cool down. The tube was then centrifuged at 4,000 g for 10 min at 4°C. The resulting supernatant was transferred to a new tube. The absorbance of the supernatant was measured at 532 and 600 nm. The content of MDA was obtained by the extinction coefficient of 155 mM^-1^ cm^-1^ at 600 nm.

### Statistical analysis

All quantification experiments were repeated three times and values were presented as the mean ± SE. All values were statistically evaluated by Student's *t*-test in a two-tailed analysis. A *P* value < 0.05 was considered to be statistically significant.

## Results

### Cloning of *IbGGPS* gene

**Fig 1:**
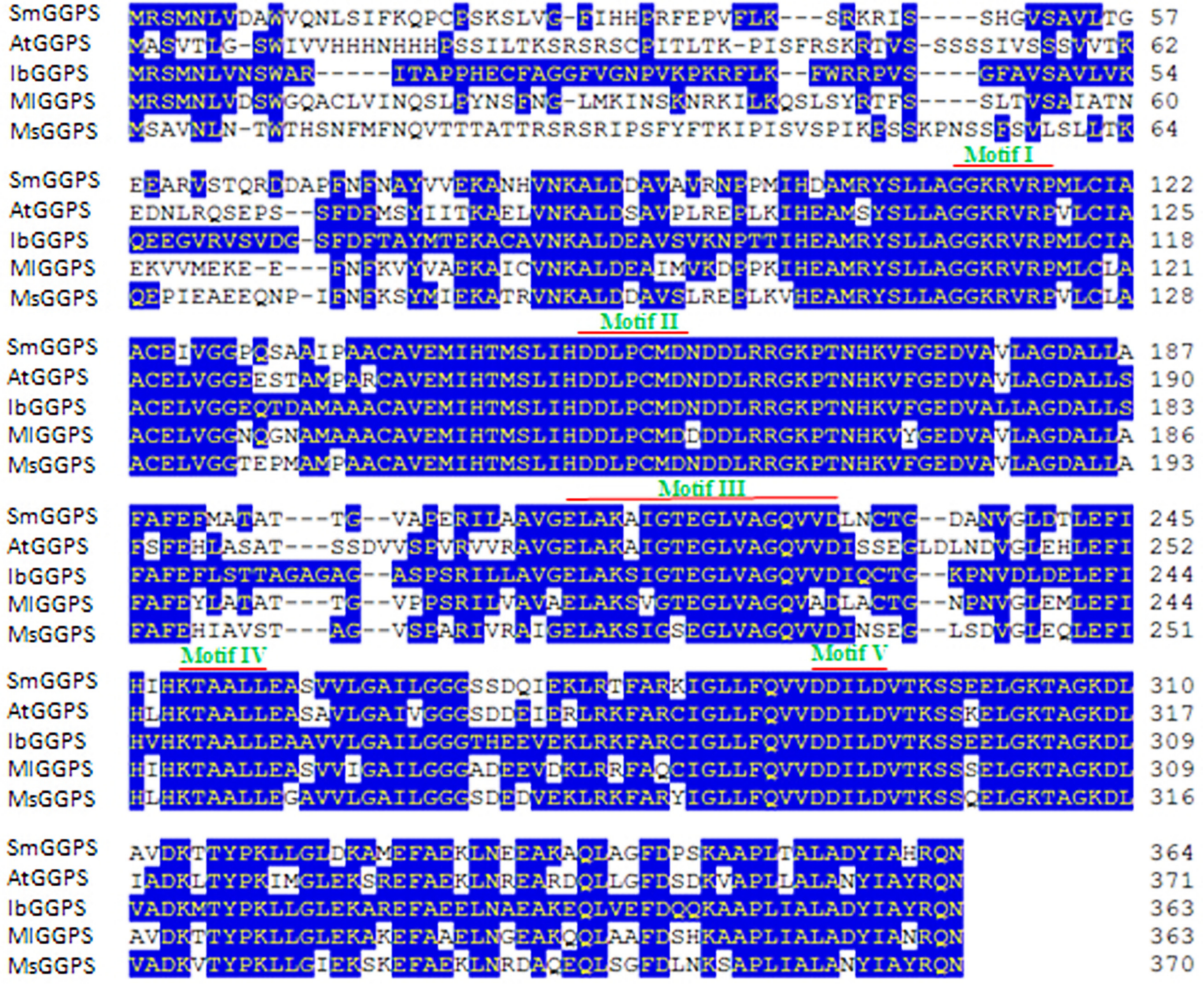
Sequence alignment of IbGGPS amino acids with five homologs. The identical and conserved amino acid residues are highlighted in blue color. Five conserved domains are designated by motif numbers (I-V). Abbreviations: IbGGPS: (KC954600); MsGGPS, *Medicago sativa* GGPS (ADG01841); SlGGPS, *Solanum lycopersicum* GPPS (NP_001234302); SmGGPS, *Salvia miltiorrhiza* GPPS (ACR19637).

**Fig 2:**
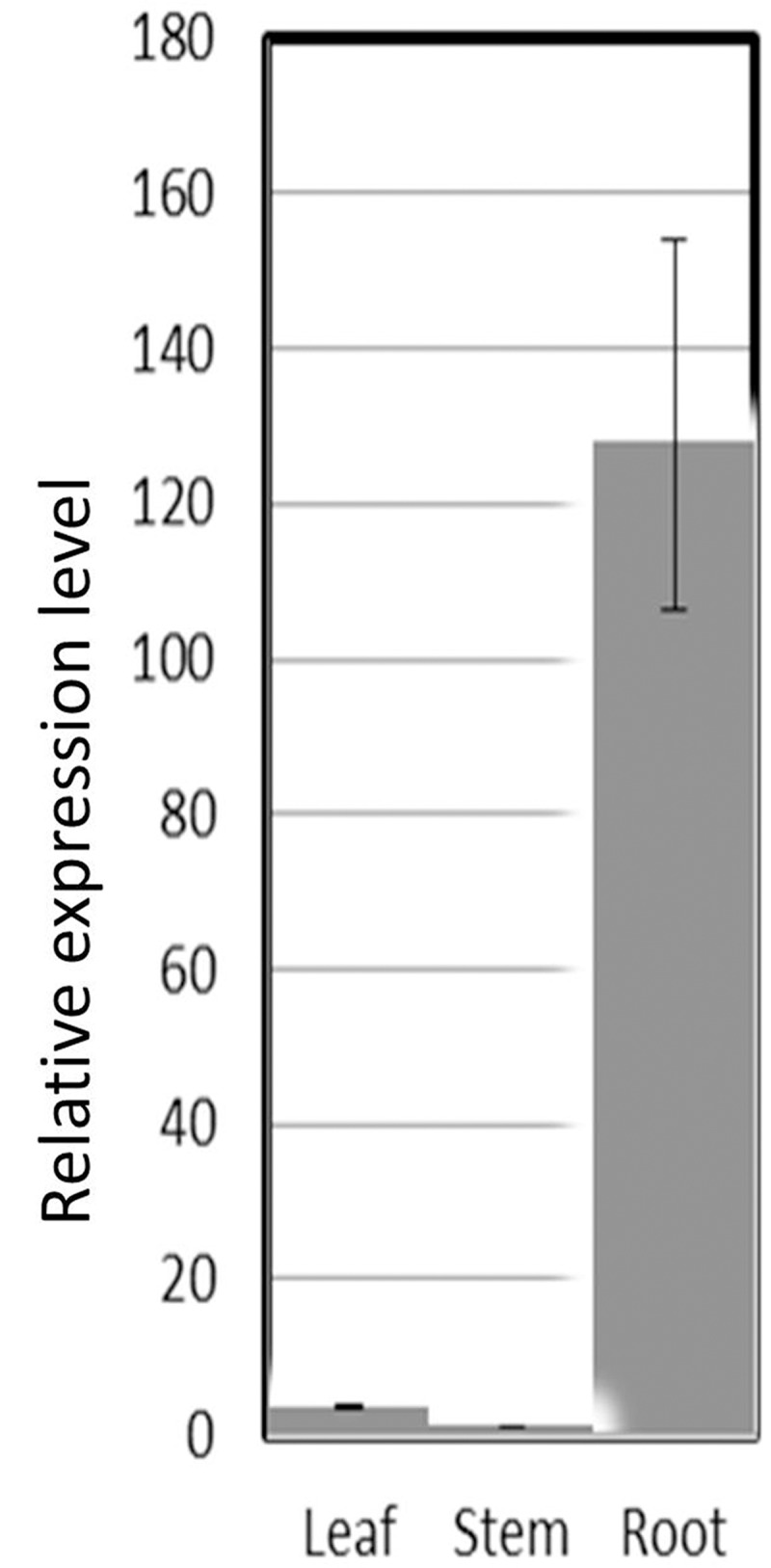
Expression comparison of the *IbGGPS* gene in leaves, stems and roots of sweetpotato. qRT-PCR analysis using the sweetpotato *β-actin* gene as an internal control was carried out to estimate expression levels. Values were obtained by denominating qRT-PCR values of stem samples. The expression level in stem is 1.0. All data were the mean values of three replicates (n = 3).

Based on the conserved domains of *GGPS* gene motif sequences in *A*. *thalliana*, *M*. *sativa*, *S*. *lycopersicum* and *S*. *miltiorrhiza*, we designed a pair of degenerate primers to isolate an *IbGGPS* gene. Using this primer pair, the fragment in the conserved region between II^nd^—and IV^th^ motives (Fig 1) was firstly amplified for sequencing. Next, the full length of cDNA including a 107 bp 5’ untranslated region (UTR) and a 70 bp 3’ UTR was amplified by RACE-PCR. To verify accuracy of the full-length cDNA sequence, two sequence specific primers were designed for amplification again. PCR and sequence analysis showed that the full length cDNA (1269-bp) contained a 1092-bp open reading frame (ORF) that encodes 363 amino acids. A multiple sequence alignment with 4 GGPS homologs showed that this amino acid sequence was 70%, 71%, 69% and 65% identical of those of *AtGGPS1*, *SmGGPS*, *SlGGPS* and *MsGGPS*, respectively (Fig 1). Particularly, the identity in the five conserved domains, I (GGKRVRP), II (DDXXXXD), III (ELAKAIGSEGLVAGQVVD), IV (KTAALL) and V (DDXXD), was extremely high (Fig 1). We designated this cDNA as *IbGGPS* (Genbank: KC954600). The same primer pair was also used to amplify genomic DNA to deduce intron-exon organization of *IbGGPS* gene. Sequencing the genomic amplicon revealed a 1542-bp sequence containing two exons (332-bp and 794-bp, respectively) and one intron (272-bp). qRT-PCR showed that the *IbGGPS* was differentially expressed in leaf, stem and root of sweetpotato. It was highly expressed in storage roots (Fig 2) but weekly expressed in stems and leaves, indicating its tissue-specificity.

### Subcellular localization of IbGGPS

**Fig 3:**
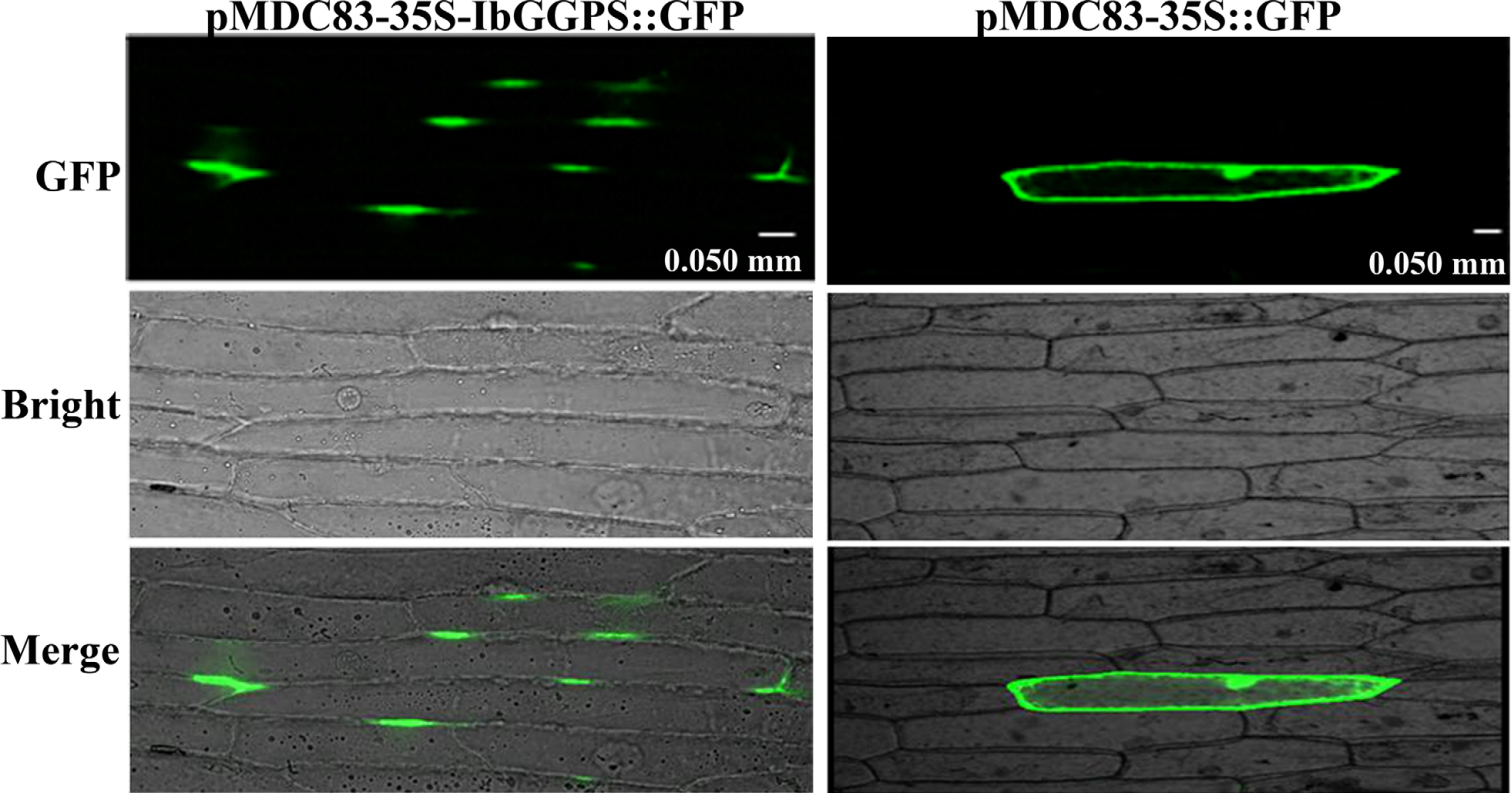
Subcellular localization of the IbGGPS::GFP fusion protein in onion epidermal cells. Confocal scanning microscopic images show localizations of IbGGPS::GFP fusion proteins in the left pMDC83-35S-IbGGPS::GFP column vs. GFP alone (as control) in the right pMDC83-35S-GFP column.

**Fig 4:**
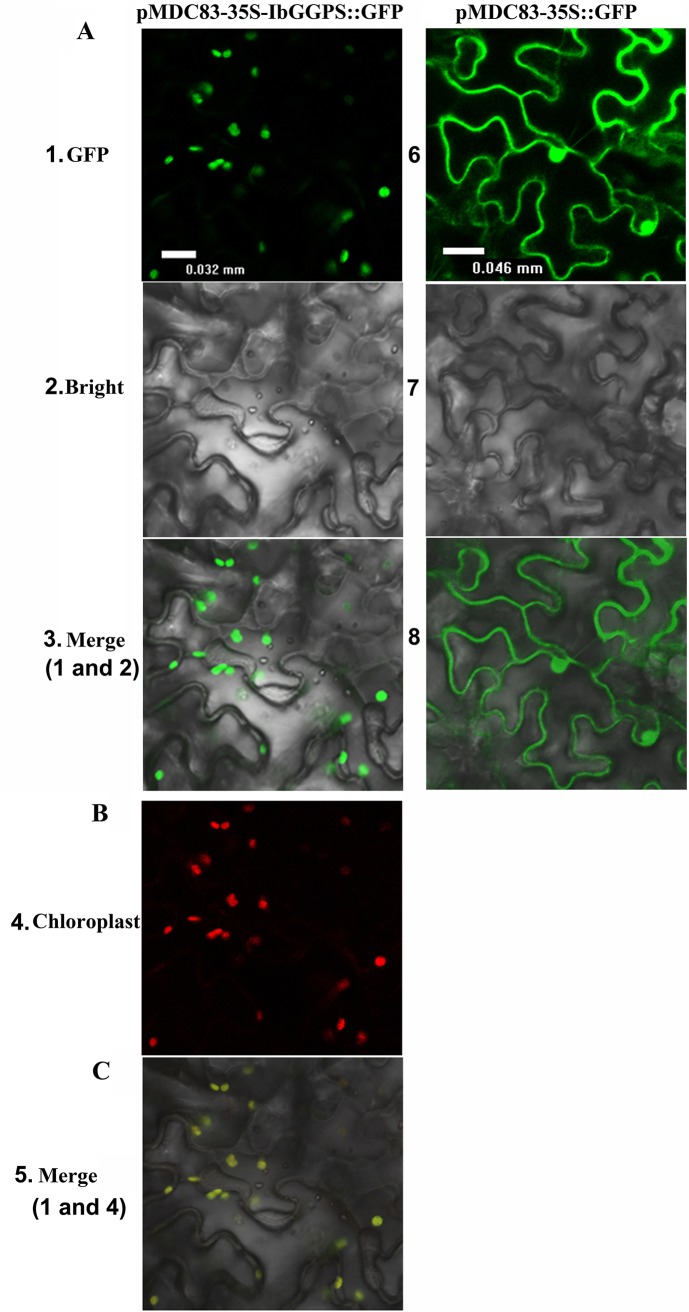
Subcellular localization of the IbGGPS::GFP fusion protein in tobacco leaf cells. (A) Confocal scanning microscopic images show localizations of IbGGPS::GFP fusion proteins in the left pMDC83-35S-IbGGPS::GFP column (images 1–3) vs GFP alone (as control) in the right pMDC83-35S-GFP column (images 6–8). (B) An image of chlorophyll autofluorescence from the same range of image 1. (C) a merged image from images 1 and 4.

Transient protein expression assays using epidermal cells of onion and leaves of tobacco were completed to reveal its subcellular localizations. Two constructs pMDC83-*IbGGPS*-GFP (fusion protein) and pMDC83-GFP (GFP alone as control) were introduced into onion epidermal cells and tobacco leaf tissues. The green fluorescence was observed using a confocal scanning microscope. The resulting images from epidermal cells of onion showed that the green fluorescence created by IbGGPS-GFP fusion protein was predominantly localized to specific areas of the plasma membrane (Fig 3). The resulting images from tobacco cells were obtained from both the green fluorescence created by IbGGPS-GFP fusion protein and the red fluorescence of chlorophyll in chloroplasts (Fig 4A–4C). The merged images showed that the IbGGPS-GFP fusion protein was localized to chloroplasts (Fig 4A–4C). By contrast, the green fluorescence of the GFP control was present throughout the cytoplasm and nucleus in both onion and tobacco leaf cells (Figs 3 and 4A).

### Overexpression of *IbGGPS* gene in *Arabidopsis thaliana*

**Fig 5:**
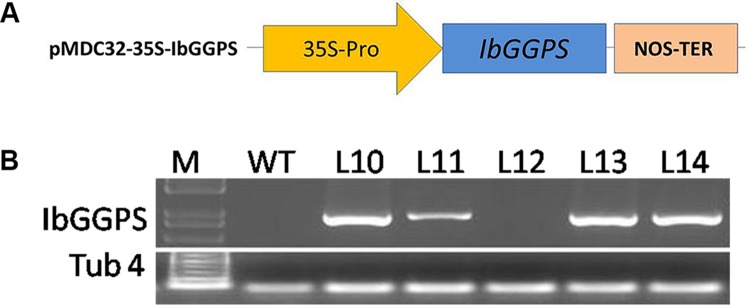
T-DNA cassette containing recombinant *35S*:*IbGGPS* (A) and a gel image of sqRT-PCR (B). Lanes L10, L11, L13 and L14: 4 transgenic *Arabidopsis* lines; Lane L12: non-transgenic *Arabidopsis* control line; Lane WT: Col-0; Lane M: 1 kb DNA marker.

**Fig 6:**
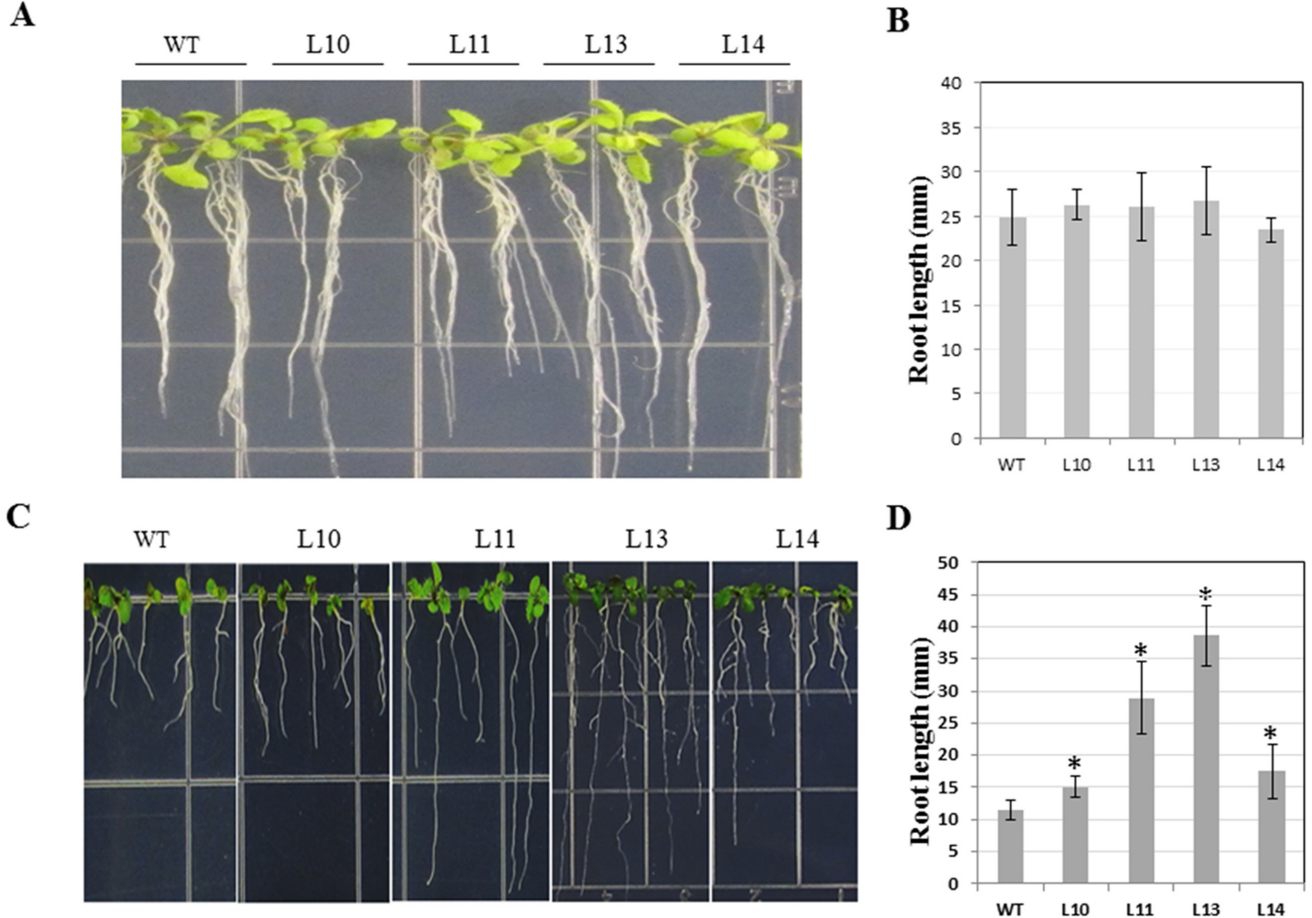
Phenotypes of transgenic vs. WT *Arabidopsis* seedlings. (A) Seedlings grown on MS medium without PEG. (B) Average root length of two-week old seedlings grown on MS medium without PEG. (C) Seedlings grown on MS medium containing PEG. (D) Average root length of two-week old seedlings grown on MS medium containing PEG. WT: Col-0; L10, L11, L13 and L14: 4 transgenic *Arabidopsis* lines. Data are presented as means ± SE (n = 3). “*” indicates a significant difference from that of WT at P < 0.05 by Student’s *t*-test.

The ORF of IbGGPS was ectopically expressed in *A*. *thaliana* (Col-0) using the binary vector pMDC32-35S-*IbGGPS* (Fig 5A). Multiple lines were obtained from Hyg resistance selection. Four transgenic lines (namely L10, L11, L13 and L14) were further demonstrated to highly express the *IbGGPS* transgene by sqRT-PCR analysis (Fig 5B), thus selected for physiological evaluation. The transgenic plants and WT showed the similar root growth on MS medium without PEG (Fig 6A and 6B). Transgenic plants were more tolerant to PEG-infused (-0.7 MPa) condition than WT. Seeds from L10, L11, L13, L14 and WT were geminated on the same PEG-infused agar-MS medium. The germination times of seeds were similar. After one week of germination, root growth differentiation phenotypes were observed among the five genotypes. The roots of the 4 transgenic lines were significantly longer than those of WT (Fig 6C and 6D). In comparison, L13 produced the longest roots (Fig 6C and 6D). In addition, L11 and L13 lines formed obviously less but longer roots on PEG-infused medium (Fig 6C and 6D) than on medium without PEG (Fig 6C and 6B). Osmotic stress might promote the root elongation in these two lines to get more water, indicating the two lines had the higher osmotic stress tolerance than WT and other transgenic lines. No other morphological or developmental changes were observed in transgenic plans.

Transgenic seedlings (L11 and L13) and WT were selected to analyze carotenoids. α-carotene, β-carotene, lutein, zeaxanthin and β-cryptoxanthin were identified from both transgenic and WT samples by HPLC. The content of total carotenoids was significantly higher in transgenic lines L11 and L13 than in WT (Table 2). In comparison, the content of α-carotene, β-carotene and lutein in transgenic plants were significant higher than in WT. By contrast, the content of β-cryptoxanthin was significantly reduced in transgenic plants than in WT (Table 2).

**Table 2:** Analysis of carotenoid content in transgenic (L11 and L13) and WT (Col-0) *Arabidopsis* lines.

Line	Lutein (μg g^-1^ FW)	Zeaxanthin (μg g^-1^ FW)	β-Cryptoxanthin (μg g^-1^ FW)	α-Carotene (μg g^-1^ FW)	β-Carotene (μg g^-1^ FW)	Total Carotenoids (μg g^-1^ FW)
**WT**	147±1.0^a^	1.37±0.021	0.998±0.270	0.328±0.098	69.6±0.6	220±0.8
**L11**	161±3.4	1.22±0.164	0.407±0.031*	1.24±0.355*	79.1±0.8*	243±2.5*
**L13**	161±9.8	1.23±0.310	0.612±0.127*	0.938±0.056*	77.3±4.4*	241±14.6*

^a^Data are presented as means ± SE (n = 3).

“*” indicates a significant difference from that of the wild-type (WT) at P<0.05 by Student’s *t*-test.

**Fig 7:**
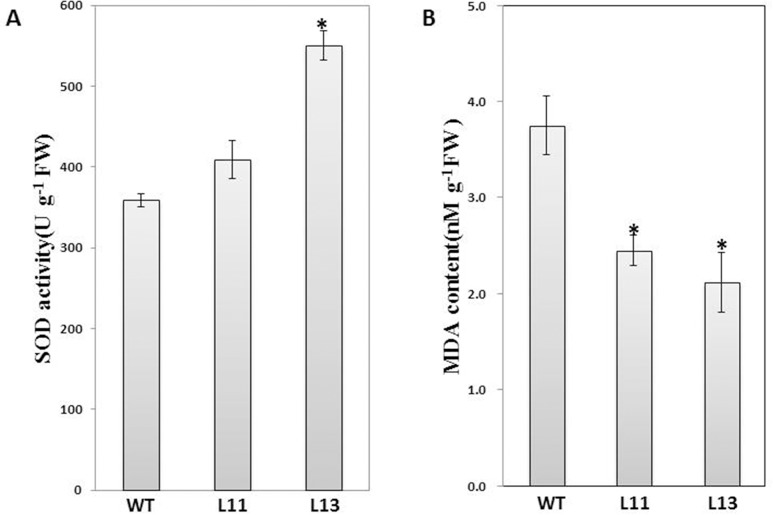
Estimation of SOD activity and MDA content in transgenic vs. WT *Arabidopsis* plants. (A) *in vitro* crude SOD activities; (B) MDA contents in leaves. L11 and L13: two transgenic lines; WT: Col-0. Data are presented as means ± SE (n = 3). “*” indicates a significant difference from that of WT at P < 0.05 by Student’s *t*-test.

The antioxidative activity of transgenic plants was evaluated by analysis of SOD activity and MDA levels. The enzymatic analysis showed that the activity of the crude SOD extraction was significantly higher in L13 than in WT and the SOD activity was also higher in L11 than in WT though the difference was not significant (Fig 7A). The measurement analysis showed that the content of MDA was significantly reduced in L11 and L13 than in WT (Fig 7B).

### Evaluation of down-stream carotenoid pathway gene expression in transgenic *Arabidopsis*

**Fig 8:**
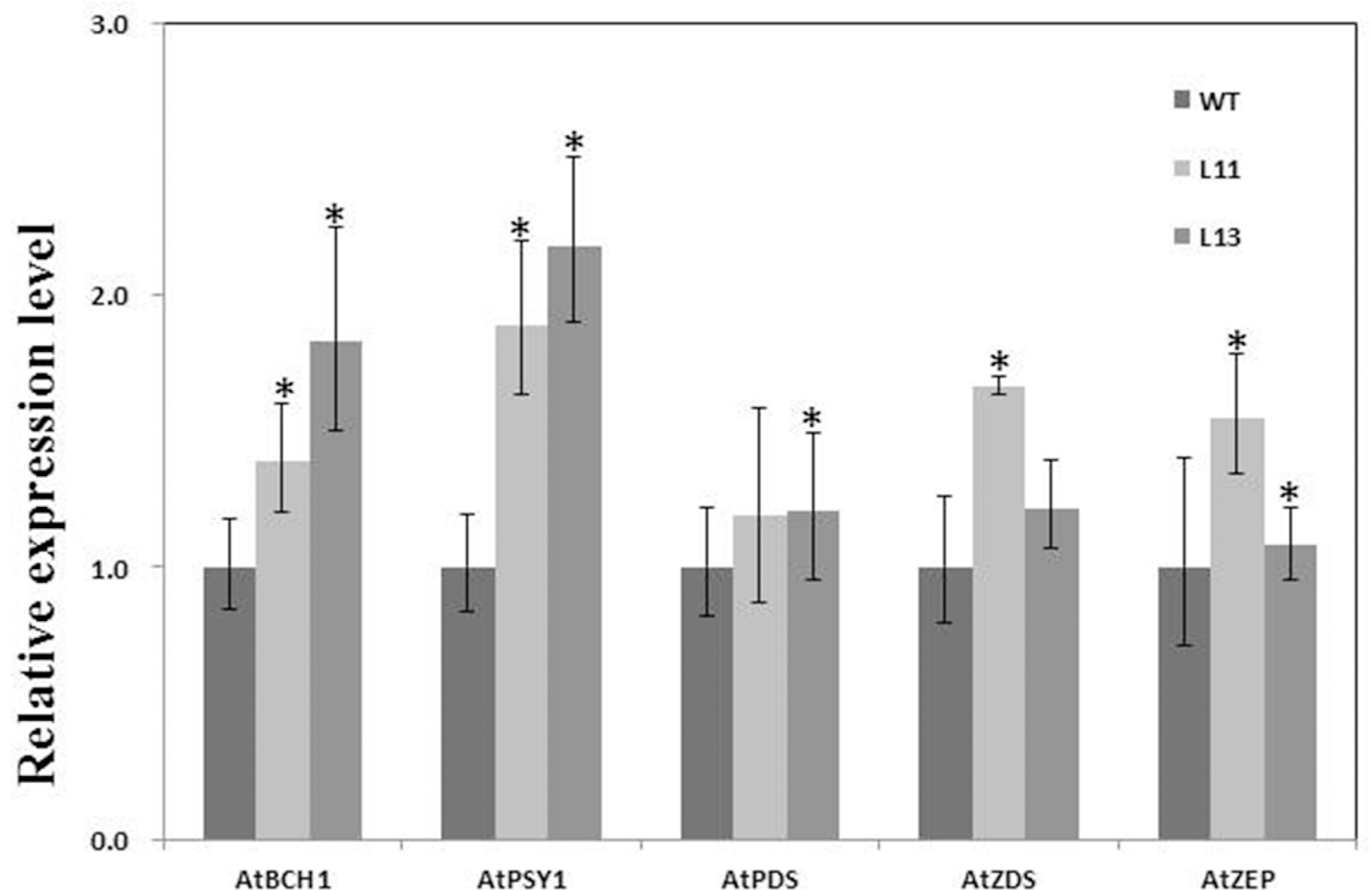
qRT-PCR analysis comparing five pathway gene expression in transgenic vs. WT plants. L11 and L13: two transgenic lines; WT: Col-0. Data are presented as means ± SE (n = 3). “*” indicates a significant difference from that of WT at P < 0.05 by Student’s *t*-test.

The expression level of *AtBCH1*, *AtPSY1*, *AtPDS*, *AtZDS* and *AtZEP* was investigated using qRT-PCR. The resulting data showed that the expression levels of *AtBCH1* and *AtPSY1* in L11 and L13 were significantly higher than in those of WT plants (Fig 8). In addition, the expression levels of *AtPDS*, *AtZDS* and *AtZEP* were increased with different patterns in L11 and L13 seedlings.

## Discussion

An *IbGGPS* cDNA was cloned from storage roots of sweetpotato in our study. The deduced amino acid sequence contains domains II and IV that has been reported to bind GGPP in homologs of *A*. *thaliana*, *M*. *sativa*, *S*. *lycopersicum* and *S*. *miltiorrhiza* plants [31]. In addition, the deduced sequence of IbGGPS includes the domain V featured by an aspartate-rich motif binding diphosphate [13]. GGPS proteins from different plants have been shown to localize in the chloroplasts/ plastids, endoplasmic reticulum (ER) and mitochondria [11, 13]. AtGGPS2-GFP and AtGGPS4-GFP proteins were localized in the ER membranes of epidermal cells hypocotyl [12]. In our experiments, the IbGGPS-GFP fusion protein localization was completed using both onion epidermis and tobacco leaf cells. The results from onion demonstrated that IbGGPS-GFP was localized in the certain membrane areas of onion epidermal cells (Fig 3). The results from tobacco leaf cells showed that IbGGPS-GFP was localized in the chloroplasts (Fig 4). These data suggested its function in plastids/chloroplasts. As well understood, GGPP is derived from the methylerythritol phosphate (MEP) pathway and is the key substrate to diterpenes and tetraterpenoids synthesized in the plastids. Manipulation of the GGPS gene has been reported to alter the production of carotenoid products, such as β-cryptoxanthin, α-carotene and β-carotene [16]. In our experiments, the *IbGGPS* cDNA was overexpressed in *A*. *thaliana*. Metabolite analysis showed an increase of carotenoids and alteration of carotenoid molecule profiles (Table 2). These molecular, transgenic and metabolic analyses demonstrated that IbGGPS is functionally associated with carotenoid biosynthesis in plastids.

The expression of *IbGGPS* is likely associated with carotenoid profiles in storage roots. The expression level of *Arabidopsis* and mustard *GGPSs* was observed to associate with the carotenoids and chlorophyll contents during seedling development [32]. In bell pepper, *GGPS* transcript abundance was highly increased during chloroplast-chromoplast conversion upon fruit ripening [33]. In our experiments, gene expression analysis showed the high expression of *IbGGPS* in storage roots but low expression in stems and leaves. When *IbGGPS* was ectopically expressed in *Arabidopsis*, the total carotenoids were increased in transgenic plants (Table 2). Metabolite profiles were also altered (Table 2). These results can be explained by alterations of gene expression in the late pathway. The overexpression of *IbGGPS* in transgenic *Arabidopsis* lines changed the expression of *AtPSY*, *AtPDS*, *AtZDS*, *AtBCH* and *AtZEP* (Fig 8). The *AtPSY* gene was up-regulated about 2 folds in transgenic lines compared to WT (Fig 8). *PSY* has been reported to control the conversion of GGPP to phytoene [34]. In the PSY-RNAi transgenic lines of tomato, the GGPP was accumulated and the total carotenoid content was remarkably reduced [35]. The overexpression of a bacterial phytoene desaturase gene *crtI*, *daffodil PSY* coupled with a lycopene β-cyclasegene (*LCYB*) produced golden rice that produced β-carotene, zeaxanthin and lutein in addition to lycopene [36]. In transgenic kiwifruit (*A*. *deliciosa*) plants expressing *GGPS*, the lutein or β-carotene contents were also increased about 1.3 folds [37]. In addition to *AtPSY*, a slight increase in *AtBCH* and *AtZDS* gene expression was observed in transgenic *Arabidopsis* lines (Fig 8), which was likely relating to carotenoid changes. The production of downstream trans-lycopene from phytoene requires reactions catalyzed by PDS, ZDS and CRTISO [38]. BCH and ZEP are related to xanthophyll biosynthesis and catalyze the production of β-carotene. The overexpression of *PDS*, *ZDS* and other downstream genes has also been reported to increase carotenoids [39].

In plants, *GGPS* genes are mainly involved in carotenoids biosynthesis [32, 35, 37]. The expression of *GGPS* was associated with protection against photo-oxidative stress in *Arabidopsis thaliana* [9]. However, there is no report about the relationship of the expression of *GGPS* and the growth of roots or osmotic stress. It was interesting that the overexpression of *IbGGPS* increased the tolerance of transgenic plants to a PEG-infused osmotic stress condition. Transgenic seedlings but not non-transgenic control plans grew longer roots (Fig 6). One mechanism was likely associated with increase of antioxidative activities in transgenic roots. Osmotic stress can increase endogenous oxidative species, such as MDA [40]. Our data showed an increase of SOD activity and a reduction of MDA content (Fig 7). The other unknown mechanism is likely associated with abscisic acid (ABA) signal. In our transgenic plants, carotenoids are increased. As well known, carotenoids are precursor molecules for ABA biosynthesis. In *Arabidopsis*, carotenoid content has been reported to positively correlate with ABA level [41]. In rice, mutations in the major genes associated with the carotenoid biosynthesis pathway resulted in ABA-deficient mutants were found to cause pre-harvest sprouting [38]. In Poaceae, the expression of *PSY3* was reported to involve a strong relationship between carotenoid content, ABA accumulation and stress response [42]. Our data will be instructional to future sweetpotato breeding for high stress-tolerant varieties.

In addition, the four transgenic *Arabidopsis* lines showed strong transcript level (Fig 5B), while there was no transcript of *IbGGPS* in WT. This high transcript level of *IbGGPS* revealed the consistent relationship to carotenoid content, root length and antioxidative activities in transgenic lines. However, there was also some inconsistence between the transcript level of *IbGGPS* and carotenoid content and root length. This may be because overexpression of *IbGGPS* causes the different patterns of expression of the downstream genes such as *AtBCH1*, *AtPSY1*, *AtPDS*, *AtZDS* and *AtZEP* in transgenic *Arabidopsis* lines (Fig 8) [37, 39].

In conclusion, an *IbGGPS* gene was cloned from storage roots of sweetpotato. Subcellular localization, transgenic approach and metabolite analysis demonstrated its involvement in the biosynthesis of carotenoids. Transgenic plants showed its expression associated with an osmotic stress tolerance. These data will be instructional to future sweetpotato breeding for high production of carotenoids and stress-tolerant varieties.
